# Caffeic acid mitigates myocardial fibrosis via suppressing MAPKs/CREB-mediated inflammation in myocardial ischemia–reperfusion injury mice

**DOI:** 10.1186/s13020-026-01562-2

**Published:** 2026-10-29

**Authors:** Mi Xiang, Xiaohu Ouyang, Shuxin Song, Mengling Yang, Alexey Sarapultsev, Shanshan Luo, Yalan Dong, Desheng Hu

**Affiliations:** 1 Department of Integrated Traditional Chinese and Western Medicine, Union Hospital, Tongji Medical College, Huazhong University of Science and Technology, No. 1277 Jiefang Ave, Wuhan, 430022 China; 2 Russian-Chinese Education and Research Center of System Pathology, South Ural State University, 76 Lenin Prospekt, Chelyabinsk, 454080 Russia; 3 Institute of Immunology and Physiology, Ural Branch of the Russian Academy of Science, 106 Pervomaiskaya Street, Yekaterinburg, 620049 Russia; 4 Institute of Hematology, Union Hospital, Tongji Medical College, Huazhong University of Science and Technology, Wuhan, 430022 China; 5 China-Russia Medical Research Center for Stress Immunology, Union Hospital, Tongji Medical College, Huazhong University of Science and Technology, Wuhan, 430022 China

**Keywords:** Caffeic acid, Myocardial fibrosis, Myocardial ischemia–reperfusion injury, MAPKs/CREB, Inflammation

## Abstract

**Background:**

Myocardial ischemia–reperfusion injury (MIRI) represents a major form of secondary damage during reperfusion therapy, in which inflammation and myocardial fibrosis are key pathological features. Caffeic acid (CA), a natural phenolic compound abundant in dietary sources, has been implicated in cardiovascular protection; however, the mechanisms by which CA modulates post-MIRI inflammation and fibrosis remain insufficiently defined.

**Objective:**

To clarify the molecular target(s) and signaling mechanism(s) through which CA attenuates inflammation-associated myocardial fibrosis after MIRI.

**Methods:**

A LAD ligation–based murine MIRI model was used to evaluate the anti-inflammatory and anti-fibrotic efficacy of CA. A co-culture system using cardiac fibroblasts and RAW 264.7 macrophages, along with adoptive cell transfer, was applied to interrogate macrophage–fibroblast crosstalk. Transcriptomic profiling (RNA-seq) was performed to identify candidate pathways involved in CA-mediated protection. Surface plasmon resonance (SPR) assay, CETSA, immunofluorescence, and western blotting were used to assess MAPKs/CREB signaling as a putative intervention axis, and pharmacological activation of MAPKs (anisomycin) or CREB (forskolin), or MAPK14 ablation, was employed to probe pathway involvement.

**Results:**

CA improved cardiac function and alleviated inflammatory and fibrotic pathology in MIRI mice, accompanied by suppression of pro-inflammatory cytokines. Conditioned medium from LPS-stimulated RAW 264.7 cells promoted fibroblast activation, and adoptively transferred macrophages within the myocardium promoted fibrotic protein expression; both effects were attenuated by CA. RNA-seq implicated MAPK signaling in the protective mechanism of CA, and downstream analyses supported inhibition of the MAPKs/CREB axis. Notably, CA achieved effects comparable to those of MAPK14 ablation, whereas anisomycin or forskolin partially blunted the anti-inflammatory and anti-fibrotic effects of CA, consistent with functional involvement of MAPKs/CREB signaling in CA-mediated protection during MIRI.

**Conclusion:**

CA mitigates MIRI-associated myocardial fibrosis by suppressing MAPKs/CREB-mediated inflammatory signaling, supporting its potential to improve long-term post-MIRI remodeling.

**Graphic Abstract:**

**Figure:**
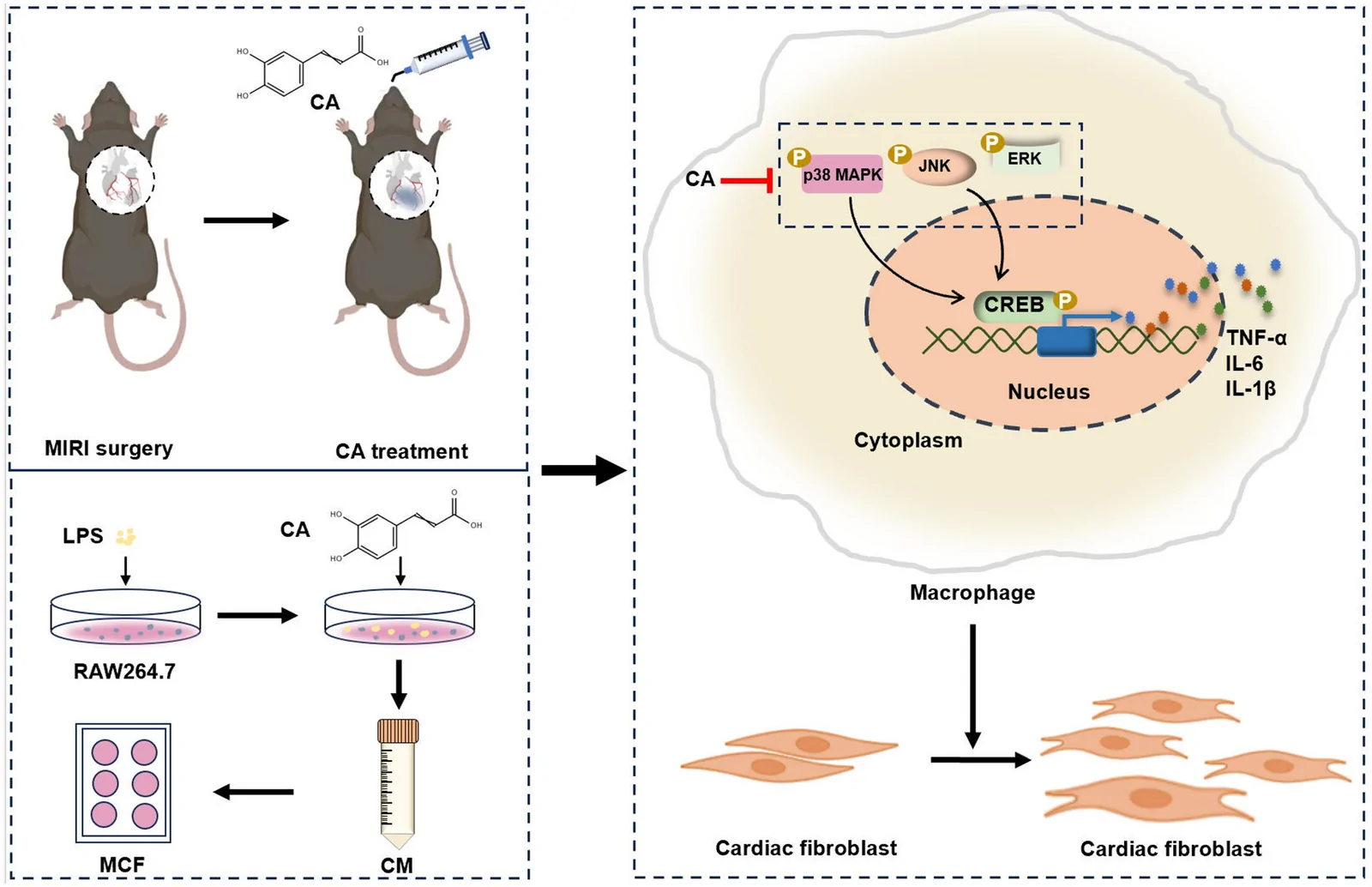

**Supplementary Information:**

The online version contains supplementary material available at https://doi.org/10.1186/s13020-026-01562-2.

## Introduction

Reperfusion is essential for salvaging ischemic myocardium; however, abrupt restoration of blood flow can paradoxically exacerbate tissue damage, a process termed myocardial ischemia–reperfusion injury (MIRI) [1, 2]. The pathophysiology of MIRI is multifactorial, involving oxidative injury, calcium overload, inflammation, and irreversible cell-death pathways, and it can culminate in cardiac dysfunction, arrhythmias, and heart failure [3, 4]. Approximately 30%–50% of patients undergoing PCI experience clinically relevant reperfusion injury, and effective strategies to mitigate this injury remain limited [3, 5].

A central pathogenic mechanism in MIRI is sterile inflammation initiated by damage-associated molecular patterns (DAMPs) [5]. Injured cardiomyocytes release endogenous danger signals that activate pattern-recognition receptors and trigger an inflammatory cascade [5]. Neutrophils and macrophages subsequently infiltrate the injured myocardium, and sustained pro-inflammatory responses contribute to adverse cardiac remodeling [6]. Although inflammation is generally viewed as part of the wound-healing response, inflammatory cell infiltration can also exacerbate tissue injury and provide fibrotic stimuli, driving pathological extracellular matrix  (ECM) deposition [7]. Myocardial fibrosis represents a common pathological hallmark of diverse cardiovascular diseases, with fibroblast activation serving as its primary driver [6, 8]. In early MIRI, cardiac fibroblasts secrete collagen to repair tissue and preserve heart structure after cardiomyocyte death; however, excessive collagen deposition increases myocardial stiffness, impairing contractility and promoting maladaptive remodeling [6, 8]. Therefore, clarifying how inflammation is coupled to fibrotic remodeling in MIRI may inform therapeutic strategies to improve long-term outcomes.

Mitogen-activated protein kinases (MAPKs), including extracellular signal-regulated kinase (ERK), c-Jun N-terminal kinase (JNK), and p38 MAPK, serve as central hubs in inflammatory signaling, transducing extracellular cues into intracellular responses that modulate gene expression and inflammatory cell behavior [9, 10]. p38 MAPK activation contributes to cardiac pathology via several mechanisms, including the induction of TNF-α and IL-6, promotion of fibroblast differentiation, and stimulation of pro-fibrotic signaling in cardiac myofibroblasts [11, 12]. CREB is a transcription factor that directly regulates genes involved in both inflammatory cytokine production and fibrotic ECM deposition [13]. Notably, p38 MAPK activation can enhance downstream CREB signaling, and the p38 MAPK/CREB axis has been implicated in cardiac fibrosis, inflammation, and pathological remodeling across various cardiovascular disease models [14, 15]. Accordingly, inhibition of the p38 MAPK/CREB axis has been associated with improvements in cardiac inflammation, fibrosis, and dysfunction [16].

Caffeic acid (CA) is a naturally occurring phenolic acid found in various dietary sources, including apples, blueberries, and cider [17, 18]. CA has been reported to exhibit multiple bioactive properties, including antioxidant, antimicrobial, and anti-inflammatory effects [17]. In vitro studies indicate that CA reduces the expression of pro-inflammatory mediators (e.g., TNF and interleukins) by modulating nuclear factor κB (NF-κB) and MAPK signaling, thereby attenuating lipopolysaccharide (LPS)-induced inflammatory injury [19]. However, systematic evidence regarding the involvement of MAPKs/CREB signaling in the inflammatory cascade and myocardial fibrosis following MIRI remains scarce, and the role of CA in this setting has yet to be explored. Therefore, we investigated whether CA attenuates inflammation-associated myocardial fibrosis in MIRI and elucidated the underlying mechanisms.

## Materials and methods

### Animal model preparation and drug treatments

Male C57BL/6 mice (7–8 weeks old; ~ 22 g) were obtained from Hubei BlONT Biological Technology Co., Ltd. (China; Approval No. SCXK(E) 2021-0027) and acclimated for 3 days. MAPK14^flox/flox^ mice (carrying loxP-flanked MAPK14 alleles) and Myh6-Cre transgenic mice (expressing Cre recombinase under the cardiomyocyte-specific myosin heavy chain 6 promoter) were obtained from Huazhong Agricultural University. These two lines were intercrossed to generate cardiomyocyte-specific MAPK14 knockout mice (MAPK14^flox/flox^; Myh6-Cre, abbreviated as MAPK14^Δ/Δ^). The MIRI model was established by transient ligation of the left anterior descending coronary artery (LAD) [20, 21]. Mice were anesthetized with intraperitoneal pentobarbital sodium (48 mg/kg) and mechanically ventilated (ALC-V8S, Shanghai Alcott Biotechnology Co., China). A left thoracotomy was performed, the heart was exposed through the third and fourth intercostal spaces, and the LAD was ligated using a 7–0 nylon suture for 30 min to induce ischemia, followed by reperfusion before chest closure. Sham-operated mice underwent the same procedure without LAD ligation. For macrophage adoptive transfer, mice received intramyocardial injections of either a cell suspension (1 × 10⁶ polarized macrophages in 50 µL sterile PBS) or sterile PBS alone, delivered at 10 µL per site across 5 sites in the LV free wall.

CA was administered by oral gavage at 12.5 mg/kg/day (MIRI+L-CA), 25 mg/kg/day (MIRI+M-CA), or 50 mg/kg/day (MIRI+H-CA). Pirfenidone was administered by gavage at 300 mg/kg/day (MIRI+Pirfenidone). On day 0, the first dose was given 10 min before MIRI modeling, followed by once-daily dosing for 14 consecutive days. Sham+veh and MIRI+veh mice received an equivalent volume of normal saline. For acute inflammatory profiling, RNA sequencing, and MAPKs/CREB pathway analyses, mice were assessed on day 3 post-MIRI, whereas cardiac function, myocardial hypertrophy, and fibrosis were evaluated on day 14 post-MIRI.

For in vivo pathway validation, anisomycin (20 mg/kg) or forskolin (2 mg/kg) was administered intraperitoneally. The first dose was given 10 min before reperfusion on day 0, followed by once-daily injections until the designated endpoint (day 3 or day 14).

Animals were housed at 24 ± 1 °C with 40%–60% relative humidity under a 12:12 h light/dark cycle, with ad libitum access to food and water.

### Cell culture and drug treatments

RAW 264.7 macrophages were obtained from Procell Life Science & Technology Co., Ltd. (CL-0190, China) and cultured in DMEM (G4511, Servicebio, China) supplemented with 10% FBS (10099141, Gibco, USA) and 1% penicillin–streptomycin (G4003, Servicebio, China). For experiments, RAW 264.7 cells were seeded in multi-well plates at a density of 1 × 10^5 cells/mL. At 70%–80% confluence, cells were treated with CA (32 or 64 μM) in the presence of LPS (1 μg/mL; Sigma-Aldrich) for 24 h.

Mouse cardiac fibroblasts (MCFs) were purchased from GuangZhou Jennio Biotech Co., Ltd. (JNO-M0170, China) and cultured in DMEM supplemented with 10% FBS and 1% penicillin–streptomycin. All cells were maintained at 37 °C in a humidified 5% CO₂ incubator (Thermo Fisher Scientific, USA).

To establish the conditioned medium (CM) co-culture system, CM was collected from RAW 264.7 cells after stimulation with LPS (1 μg/mL) and CA (32 or 64 μM) for 24 h. The supernatant was centrifuged at 1000×*g* for 5 min to remove cell debris. MCFs were then cultured with RAW 264.7-derived CM and subjected to subsequent assays.

To address whether CA exerted direct anti-fibrotic effects upon activated fibroblasts in the absence of inflammatory cells, we performed an in vitro experiment in which cardiac fibroblasts were treated with TGF-β1 (10 ng/mL) to induce activation, with or without CA (64 μM) for 24 h. Cells were then detected with ɑ-SMA and collagenⅢ in order to assess fibroblast activation.

### Bone marrow macrophage culture and adoptive cell transfer.

Bone marrow cells were harvested aseptically from C57BL/6 mouse femurs and tibias and cultured in RPMI 1640 medium, supplemented with 10% FBS, 1% penicillin–streptomycin and 30 ng/mL recombinant mouse macrophage-colony-stimulating factor (M-CSF) at 37 °C. The medium was replaced with fresh one containing 30 ng/mL M-CSF every 3 days for 7 days. Bone marrow-derived macrophages (BMDMs) were then stimulated with LPS (100 ng/mL) and IFN-γ (10 ng/mL) to promote M1 polarization. After 24 h of polarization, the macrophages were used for intramyocardial adoptive cell transfer into C57BL/6 mice.

### Cardiac function detection

Echocardiography was performed 2 weeks after MIRI induction or sham surgery using a Vevo3100 ultrasound system (VisualSonics Inc., Canada). Anesthetized mice were shaved over the thorax and positioned supine on a physiological monitoring platform. Limb electrodes were attached for real-time heart rate monitoring, and recordings were obtained after a stable heart rate (300–500 beats/min) was achieved. Left ventricular (LV) function was assessed using M-mode images acquired at the papillary muscle level to measure LV ejection fraction (LVEF) and fractional shortening (LVFS). Measurements were analyzed using VevoLAB software (VisualSonics Inc., Canada) by averaging at least three consecutive cardiac cycles.

### H&E staining and Masson staining

Hematoxylin and eosin (H&E) staining was performed to evaluate histopathological changes in cardiac tissue. Samples were fixed in 4% paraformaldehyde overnight, dehydrated, cleared, embedded in paraffin, and sectioned transversely. Sections were deparaffinized in xylene, rehydrated through graded ethanol, and stained with hematoxylin for 5 min. Differentiation was performed using 1% hydrochloric acid for 30 s, followed by eosin staining (1%) for 5 min. Sections were then dehydrated through graded ethanol, cleared in xylene, mounted with neutral balsam under a coverslip, and air-dried. Images were acquired using a Pannoramic Scan II slide scanner (3DHISTECH Ltd., Hungary).

Masson’s trichrome staining was performed to assess myocardial fibrosis. After deparaffinization and rehydration as described above, sections were stained with Weigert’s iron hematoxylin for 8 min. Following brief differentiation in acid alcohol, sections were stained with 0.7% Masson–Ponceau S acid fuchsin (S0072, Bioss, China) for 10 min and rinsed with 2% pre-cooled acetic acid. Sections were then treated with phosphomolybdic acid for 2 min and rinsed again with acetic acid, followed by staining with 2% aniline blue. After a final acetic acid rinse, sections were dehydrated, cleared, and mounted. Slides were scanned using a Pannoramic Scan II system.

### Immunofluorescence staining

Tissue sections were deparaffinized and rehydrated, followed by antigen retrieval in boiling sodium citrate buffer (SL1870, Coolaber, China) for 45 min. Sections were permeabilized in PBS containing 0.1% Triton X-100 for 20 min. Non-specific binding was blocked with 5% normal goat serum for 1 h at room temperature. Sections were stained with a diluted antibody solution mixed with rabbit-derived CD86 (ER1906-01, HUABIO, China) and mouse-derived CD206 (HA601453, HUABIO, China), stained with a solution mixed with rabbit-derived CD86 and mouse-derived α-SMA (HA601546, HUABIO, China), or stained with fluorescein-conjugated wheat germ agglutinin (WGA) lectin (GTX01501, Gene Tex, USA). The sections been incubated with CD86 and CD206, or CD86 and α-SMA, were then incubated with a solution mixed with iFluor™ 488 conjugated goat anti-rabbit IgG (HA1121, HUABIO, China) and iFluor™ 594 conjugated goat anti-mouse IgG (HA1126, HUABIO, China). Slides were mounted using SuperKine™ enhanced anti-fade mounting medium containing DAPI and scanned with an automated slide scanning system (Olympus, Japan).

RAW 264.7 macrophages were seeded in confocal dishes. After attachment, cells were treated with LPS (1 μg/mL) and CA (64 μM) for 24 h. Cells were then fixed, permeabilized, and blocked, followed by incubation with a phospho-CREB primary antibody (1:200, ET7107-93, HUABIO, China) overnight at 4 °C. After washing, cells were incubated with Cy3-labeled goat anti-rabbit IgG (A0516, Beyotime, China) for 1 h in the dark, mounted using SuperKine™ enhanced anti-fade mounting medium containing DAPI, and imaged using a confocal microscope (Nikon, Japan).

### Flow cytometry analysis

Immediately after euthanasia, hearts were perfused with PBS, excised, and rinsed in PBS to remove residual blood. Cardiac tissue was minced and digested in 3 mL HBSS containing collagenase II (1 mg/mL; LS004176, Worthington, USA) at 37 °C for 30 min with continuous agitation. Digestion was stopped by adding RPMI 1640 (G4531, Servicebio, China) supplemented with 10% FBS (10099141, Gibco, USA). The cell suspension was filtered and centrifuged to collect cells. Cells were incubated at 4 °C for 30 min in the dark with fluorophore-conjugated anti-mouse antibodies: CD45-FITC (clone 104), Ly6C-PE (clone AL-21), CD11c-PE/Cy7 (clone HL3), CD19-APC (clone 6D5), CD3-APC (clone 145-2C11), Ly6G-APC-Cy7 (clone 1A8), F4/80-BV421 (clone T45-2342), and CD11b-BV510 (clone M1/70) (all from BD Biosciences, USA). Dead cells were excluded using Fixable Viability Stain 510 (564406, BD, USA). Cells were washed, resuspended in PBS, filtered through a 200-mesh strainer, and analyzed on a CytoFLEX flow cytometer. Data were acquired using CytExpert software (v2.5, USA).

### RNA sequencing and bioinformatics analysis

Heart tissues were harvested at day 3 post-MIRI. Total RNA was extracted using TRIzol™ reagent (15596018CN, Invitrogen, USA) according to the manufacturer’s instructions. RNA concentration and purity were assessed using a NanoDrop spectrophotometer (Thermo, USA). Sequencing libraries were prepared and analyzed on the BGISEQ-500 platform (BGI, China). Raw reads were quality-filtered to generate clean data, which were aligned to the mouse reference genome (Mus musculus GRCm38.p5, NCBI) using HISAT2 (v2.2.1). Gene expression was quantified, and differential expression analysis was performed using the DESeq2 R package with a significance threshold of P < 0.05 and |log2 fold change|≥ 1 (fold change ≥ 2). Gene Ontology (GO) and KEGG pathway enrichment analyses were performed and visualized using the clusterProfiler package in RStudio. To evaluate gene connectivity, the union gene list was submitted to the STRING database to generate a protein–protein interaction (PPI) network, and interactions were visualized in Cytoscape (v3.9.1, USA).

### Transwell migration assay

Transwell inserts (24-well; 8-μm pore size; PTEP24H48, Merck, Germany) were used to assess cell migration. MCFs were suspended in RAW-CM (1 × 10^5 cells/mL), and 200 μL of the cell suspension was added to the upper chamber. The lower chamber was filled with medium supplemented with 20% FBS. After 24 h incubation, cells on the upper chamber were fixed with 4% paraformaldehyde for 15 min and stained with crystal violet for 15 min. Following removal of the non-migratory cells from the upper surface, the migrated cells on the lower membrane surface were imaged using a microscope (CKX53, Olympus, Japan), and migration was quantified by counting cells in three randomly selected fields per insert.

### Wound healing assay

MCFs were seeded in 6-well plates. After attachment, culture medium was replaced with RAW-CM, and cells were incubated until 95%–100% confluence. A scratch wound was generated using a sterile pipette tip (1250 μL). Cells were gently washed with sterile PBS and cultured in CM for an additional 12 h. Wounds were imaged at 0 and 12 h, and wound width was quantified using ImageJ (NIH, USA).

### 5-Ethynyl-2′-deoxyuridine (EdU) assay

Coverslips were placed in 24-well plates before seeding MCFs. When cells cultured in RAW-CM reached 70%–80% confluence, the EdU assay was initiated. After treatments, cells were labeled by adding pre-warmed (37 °C) 2× EdU working solution (20 μM) to the existing medium at a 1:1 ratio and incubating for 2 h. Cells were then fixed with 4% paraformaldehyde for 15 min at room temperature, washed, and permeabilized with 0.3% Triton X-100 in PBS for 12 min. After washing, the click reaction was performed by adding 200 μL reaction cocktail per well and incubating for 30 min at room temperature in the dark. Coverslips were mounted using anti-fade mounting medium containing DAPI for fluorescence imaging.

### Western blot

Proteins were extracted from heart tissue, MCFs and RAW 264.7 cells using RIPA lysis buffer (G2002, Servicebio, China) supplemented with 1% phenylmethanesulfonyl fluoride (PMSF; G2008, Servicebio, China) and 1% phosphorylated protease inhibitor (G2007, Servicebio, China). Protein samples were separated by SDS-PAGE and transferred to PVDF membranes (ISEQ00010 or IPVH00010, Merck, Germany). Membranes were blocked with bovine serum albumin (V900933, Merck, Germany) for 1 h at room temperature and incubated with primary antibodies overnight at 4 °C. Primary antibodies included: α-SMA (1:2000, ET1607-43, HUABIO, China), collagen III (1:1000, HA720050, HUABIO, China), phospho-p38 MAPK (Thr180/Tyr182) (1:1000, 28796-1-AP, Proteintech, China), p38 MAPK14 (1:1000, ET1702-65, HUABIO, China), phospho-ERK1 (T202/Y204) + ERK2 (T185/Y187) (1:2000, ET1610-13, HUABIO, China), ERK1/2 (1:500, T40071F, Abmart, China), phospho-JNK1/2/3 (T183/T183/T221) (1:1000, ET1609-42, HUABIO, China), JNK1/2/3 (1:1000, T40073F, Abmart, China), phospho-CREB (S133) (1:1000, ET7107-93, HUABIO, China), CREB (1:500, ET1601-15, HUABIO, China), PCNA (1:5000, A12427, ABclonal, China), CCND1 (1:1000, A19038, ABclonal, China), vinculin (1:1000, GB111328, Servicebio, China), and α-actin (1:1000, 23660-1-AP, Proteintech, China). Membranes were then incubated with HRP-conjugated anti-rabbit IgG (1:10,000, HA1001, HUABIO, China) for 1 h at room temperature. Signals were detected using an iBright™ CL750 imaging system (Invitrogen, USA) with ECL substrate (WBULS0100, Merck, Germany). Band intensities were quantified using ImageJ.

### Molecular docking

The three-dimensional structure of CA was downloaded from the PubChem database (https://pubchem.ncbi.nlm.nih.gov/) and converted by the science codons database (https://sciencecodons.com/tools/sdf-to-pdb-converter/). The molecular structure of MAPK was gained from the protein data bank database (https://www.rcsb.org/). Both MAPK and ligand molecules were prepared using AutoDock Tools (version 1.5.7). A standard protein preparation workflow was used to hydrogenate, calculate Gasteiger charges, minimize energy, and obtain the final optimized ligand structures. The AutoDock tools was used to set grid center point dimensions at X = 49.516, Y = 66.776, Z = 22.554. The box dimensions (Å) were set at X = 70.567, Y = 76.65, and Z = 55.967. The final docking was performed using the AutoDock Vina, following by the optimal interaction conformation selected based on binding poses and affinity scores. The obtained result was visualized utilizing PyMol (version 3.1.6.1).

### Surface plasmon resonance (SPR) assay

The binding affinity and kinetic parameters of human p38 α/MAPK14 protein (HY-P73335, MedChemExpress, USA) to CA was evaluated by SPR (Biacore 8 K, GE Healthcare) using established protocols. The protein (50 μg/mL in acetate buffer) was amine-coupled to CM5 sensor chips via EDC/NHS activation, followed by blocking with ethanolamine. Another channel served as the reference and was processed identically, except that acetate buffer without protein was used during the immobilization step. Analytes were serially diluted and injected over the chip from low to high concentrations at 30 μL/min for 60 s per cycle. The chip was regenerated with 10 mM glycine–HCl (pH 2.0) for 5 min between runs. Data were globally fitted to a 1:1 Langmuir binding model using Biacore Insight software (Cytiva, USA) to derive the following kinetic parameters: the association rate constant (ka), dissociation rate constant (kd), and dissociation constant (KD).

### Cellular thermal shift assay (CETSA)

RAW 264.7 macrophages were incubated with DMSO or CA (64 μM) for 2 h. Total proteins were extracted using immunoprecipitation lysis buffer for 30 min. Lysates were aliquoted into seven PCR tubes and heated at 40, 45, 50, 55, 60, 65, and 70 °C for 10 min. After centrifugation at 12,000×*g* for 10 min, supernatants were collected and analyzed by western blotting. Band intensities were quantified using ImageJ, and CETSA thermal stability curves were generated using GraphPad Prism (v9.5.0, USA).

### Enzyme-linked immunosorbent assay (ELISA)

Blood was collected from the retro-orbital venous plexus under anesthesia using a capillary tube. Serum was obtained by centrifugation at 3000×*g* for 15 min at 4 °C for subsequent cytokine measurements. For in vitro assays, RAW 264.7 cells were treated with LPS (1 μg/mL) with or without CA (64 μM) and the MAPK agonist anisomycin (5 μM) for 24 h; supernatants were collected to assess cytokine production. IL-6 (E-EL-M0044, Elabscience, China), TNF-α (E-EL-M3063, Elabscience, China), and IL-1β (E-EL-M0037, Elabscience, China) levels in serum and culture supernatants were quantified using ELISA kits according to the manufacturers’ instructions.

### Quantitative real time polymerase chain reaction (RT-qPCR)

At the experimental endpoint, heart tissue (in vivo) and RAW 264.7 macrophages or MCFs (in vitro) were harvested. Total RNA was extracted using TRIzol™ reagent (15596018CN, Invitrogen, USA), followed by chloroform extraction, isopropanol precipitation, ethanol washing, and dissolution in DEPC-treated water. RNA concentration and purity were assessed using a NanoDrop spectrophotometer, and RNA was reverse-transcribed into cDNA. RT-qPCR was performed using a CFX Connect™ Real-Time System (BIO-RAD, USA) with 2× Universal SYBR Green Fast qPCR Mix (RK21203, ABclonal, China). Cycling conditions were: 95 °C for 3 min, followed by 40 cycles of 95 °C for 5 s and 60 °C for 30 s. Relative expression levels were calculated using the 2^−ΔΔCt method after confirmation of amplification specificity by melt-curve analysis. α-Actin was used as an internal reference gene. Primer sequences are listed in Table 1.

**Table 1:** Primer sequences for quantitative real time polymerase chain reaction

Species	Gene	Forward	Reverse
Mouse	*Il1β*	TCTAGGCTTTCAATGAGTGTGCC	ATCTTTTGGGGTCCGTCAACT
Mouse	*Tnfα*	CCCTCACACTCAGATCATCTTCT	GCTACGACGTGGGCTACAG
Mouse	*Il6*	TCACAGAAGGAGTGGCTAAGGACC	ACGCACTAGGTTTGCCGAGTAGAT
Mouse	*Inos*	GTTCTCAGCCCAACAATACAAGA	GTGGACGGGTCGATGTCAC
Mouse	*Arg1*	AGGGTCCACCCTGACCTATG	ACAGGTTGCCCATGCAGATT
Mouse	*Ccnd1*	TCAAGTGTGACCCGGACTG	ATGTCCACATCTCGCACGTC
Mouse	*Ccnd2*	GGGAACTGGTAGTGTTGGGTAAG	AATCATCGACGGCGGGTAC
Mouse	*Ccnd3*	TGGAGAACGCCTTCCTGAAC	CCCTTGGTGTCTTGCTGGAT
Mouse	*Pcna*	AAAGATGCCGTCGGGTGAAT	TGGTTACCGCCTCCTCTTCT
Mouse	*α-actin*	GAGGCACCACTGAACCCTAA	CATCTCCAGAGTCCAGCACA

### Statistical analysis

Data are representative of at least three independent biological replicates (in vitro) or were derived from cohorts of at least three animals per group (in vivo). For comparisons between two groups, Student’s t-test was used for normally distributed data, and the Mann–Whitney U test was used for non-normally distributed data. For comparisons among multiple groups, one-way ANOVA was applied. In the sequencing analysis, the Wilcoxon rank-sum test was used for two-group comparisons. Data are presented as mean ± SD as appropriate, and P < 0.05 was considered statistically significant. GraphPad Prism (v9.5.0, USA) was used for statistical analyses and figure generation.

## Results

### Caffeic acid ameliorated cardiac fibrosis and dysfunction in MIRI mice

**Fig. 1:**
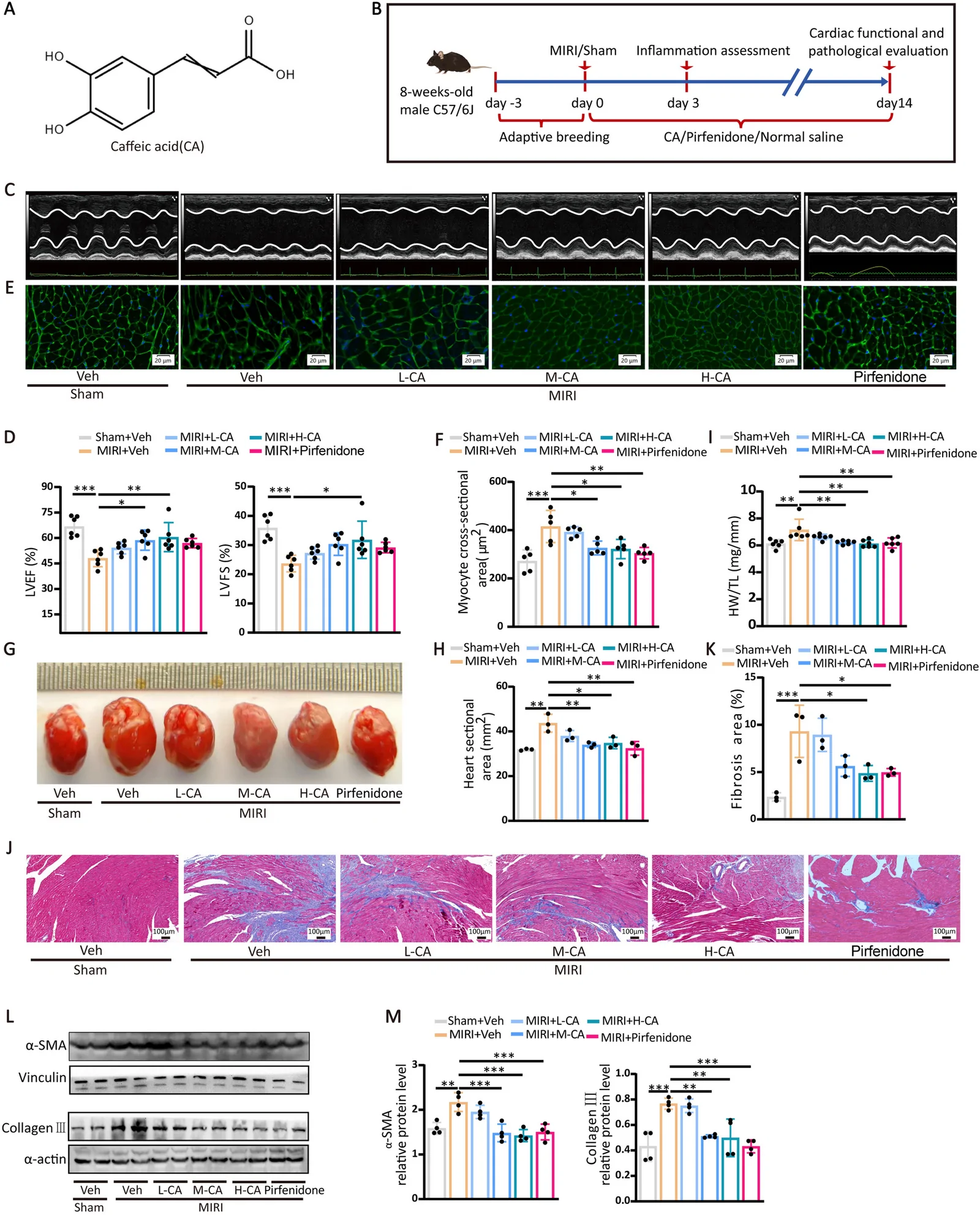
Caffeic acid improved cardiac dysfunction and fibrosis in MIRI-induced mice. **A** Two-dimensional chemical structure of CA. **B** Schematic diagram of the experimental protocol. MIRI models or control models were established by LAD ligation or by the same surgical procedure without ligation. Starting 10 min before MIRI modeling (day 0) and continuing until day 14, different doses of CA (12.5 mg/kg, 25 mg/kg, 50 mg/kg), pirfenidone (300 mg/kg) or an equivalent volume of normal saline were administered once daily by oral gavage. Inflammatory markers, cardiac function, and pathological changes were assessed at designated time points. **C** Representative short-axis M-mode echocardiographic images at 2 weeks after MIRI. **D** Corresponding quantitative measurements of LVEF and LVFS. n = 6 mice per group). **E** Morphological change of cardiomyocytes as shown by staining with WGA. Scale bar = 20 μm. **F** Measurements of cardiomyocytes size (green-stained) based on WGA staining. Cardiomyocytes were outlined in transversely sectioned images using ImageJ software, and the mean area was calculated from at least 20 cells per sample. n = 5 mice per group. **G** Images of the hearts from mice when the study terminated. **H** Quantitative measurements of longitudinal cardiac section using ImageJ software. n = 3 mice per group. **I** Ratios of heart weight-to-tibial length. n = 6 mice per group. **J** Masson's trichrome staining of myocardial sections. Myofibers and collagen are shown in red and blue, respectively. Scale bar = 100 μm. **K** Quantitative analysis of fibrosis. The fibrosis area (%) in the infarct border zone was calculated using ImageJ by measuring the ratio of Masson-positive (blue) area to the total field area in randomly selected fields. n = 3 mice per group. **L**, **M** Protein analysis of α-SMA and collagen III in the myocardium of mice through western blot analysis. Band intensities were quantified using ImageJ software. Vinculin and α-actin served as loading controls. n = 4 mice per group. *P < 0.05, **P < 0.01, ***P < 0.001. Statistical analyses were performed using one-way ANOVA followed by Tukey's post hoc test. Results are presented as mean ± SD

CA was administered by oral gavage at 12.5, 25, or 50 mg/kg/day and pirfenidone was at 300 mg/kg/day; the first dose was given 10 min before MIRI modeling on day 0, followed by once-daily dosing for 14 days (Fig. 1B). Cardiac function was first assessed by echocardiography. Representative M-mode images are shown in Fig. 1C. MIRI markedly reduced LVEF and LVFS, whereas CA treatment (50 mg/kg/day; H-CA) improved both parameters in MIRI mice (Fig. 1D). Cardiomyocyte morphology was evaluated by WGA staining: cardiomyocytes were enlarged in the MIRI+veh group compared with sham controls, and this hypertrophic change was attenuated by medium- and high-dose CA and pirfenidone (Fig. 1E, F). Consistently, gross examination revealed cardiac enlargement after MIRI, which was alleviated by medium- and high-dose CA and pirfenidone (Fig. 1G, H), and this was supported by a reduced heart weight-to-tibial length (HW/TL) ratio (Fig. 1I). Given that myocardial fibrosis contributes to LV dysfunction and progression to heart failure, fibrotic remodeling was assessed by Masson’s trichrome staining. MIRI induced prominent collagen deposition and increased fibrotic area, both of which were substantially reduced by CA administration (Fig. 1J, K). In line with these histological findings, α-SMA and collagen III protein levels were elevated in MIRI hearts and were suppressed by CA treatment (Fig. 1L, M). Furthermore, the decreases in myocardial fibrotic scar area and fibrotic protein expression after CA treatment were comparable to those observed in the pirfenidone-treated group. Collectively, these results indicate that CA improves cardiac function and attenuates cardiac hypertrophy and myocardial fibrosis in MIRI mice.

### Caffeic acid suppressed post-MIRI cardiac inflammation

**Fig. 2:**
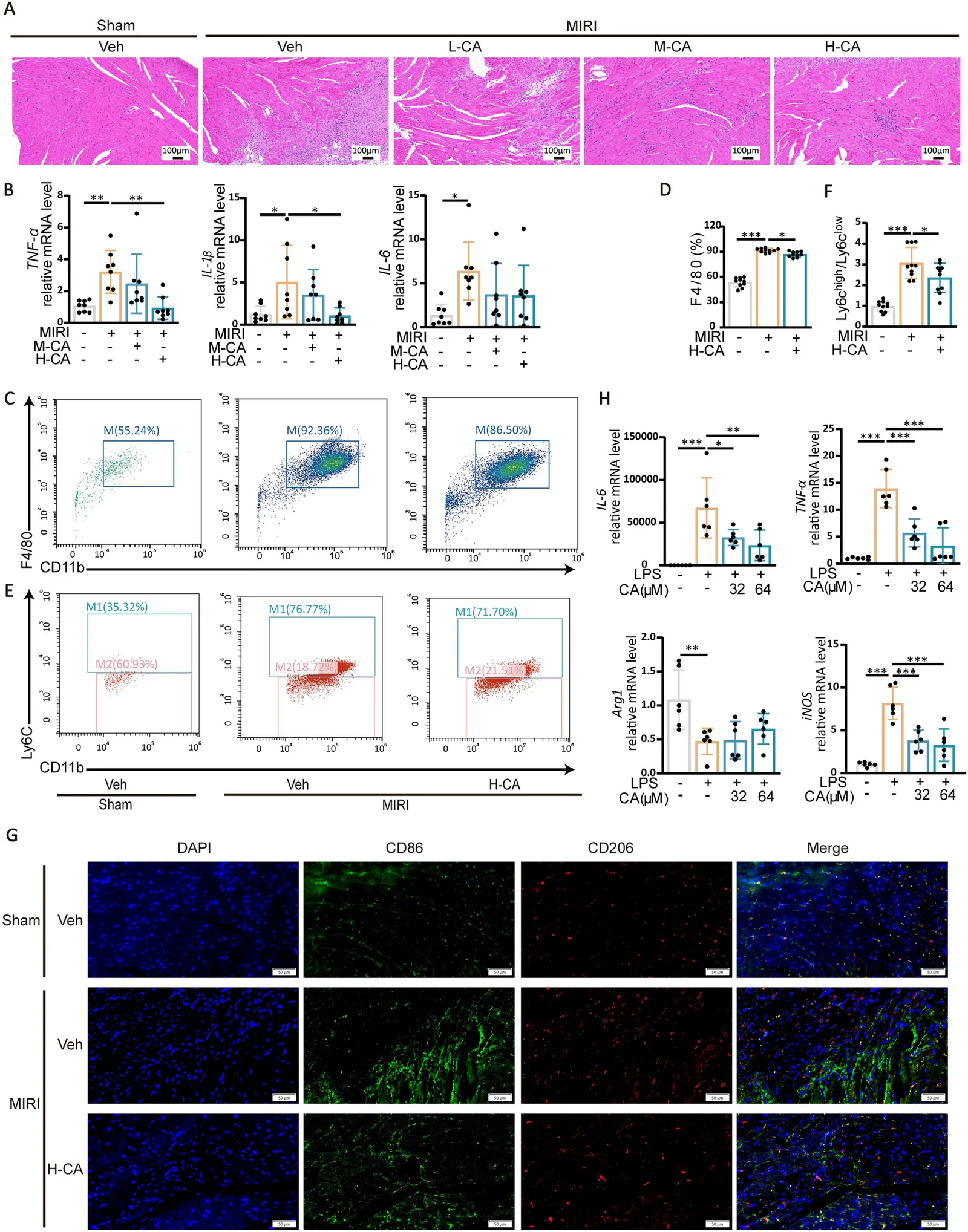
Caffeic acid improved myocardial injury and inflammatory response in MIRI-induced mice. **A** H&E staining of myocardial sections. View fields were randomly selected in the infarct border zone. Scale bar = 100 μm; n = 3 mice per group. **B** Relative mRNA levels of TNF-α, IL-1β, and IL-6 in heart tissue. n = 8 mice per group. **C**, **D** Flow cytometry to detect macrophage frequency. n = 10 mice per group. **E**, **F** Flow cytometry to detect the phenotypic changes of macrophages. n = 10 mice per group. **G** Representative immunofluorescence images showing CD86 and CD206 expression on cardiac tissue. Green, CD86; red, CD206; blue, cell nuclei. Scale bar = 50 μm; n = 3 mice per group. **H** Relative mRNA levels of IL-6, TNF-α, Arg1, and iNOS in RAW 264.7 following LPS or CA treatment. n = 6 independent biological replicates per group. *P < 0.05, **P < 0.01, ***P < 0.001. Statistical analyses were performed using one-way ANOVA followed by Tukey's post hoc test. Results are presented as mean ± SD

Because chronic inflammation is a key driver of fibrosis [22], we next assessed cardiac inflammation in our model. H&E staining showed that cardiomyocytes were orderly arranged in the control myocardium, whereas myocardial tissue from MIRI mice exhibited necrosis, structural disorganization, and marked inflammatory cell infiltration. These pathological changes were alleviated in a dose-dependent manner by medium and high doses of CA (Fig. 2A). Consistently, CA reduced the cardiac mRNA levels of the pro-inflammatory cytokines TNF-α and IL-1β (Fig. 2B). IL-1β, IL-6, and TNF-α are key mediators that can amplify TGF-β signaling, thereby promoting fibroblast-to-myofibroblast differentiation and subsequent ECM overproduction [23–25]. Thus, the cytokine changes observed here suggest that CA may mitigate inflammation-driven myocardial fibrosis.

Because macrophage-initiated immune responses play an important role in MIRI, we quantified myocardial macrophage populations by flow cytometry. MIRI induction markedly increased F4/80⁺ macrophage infiltration, whereas CA treatment counteracted this increase in macrophage frequency (Fig. 2C, D). Pro-inflammatory and anti-inflammatory macrophages were defined as Ly6C-positive and Ly6C-negative populations, respectively [26]. In MIRI mice, the Ly6C⁺ population increased significantly, consistent with a shift toward a pro-inflammatory macrophage phenotype (Fig. 2E, F). CA treatment attenuated this phenotypic shift (Fig. 2E, F), supporting its anti-inflammatory effect. Subsequently, we performed co-immunostaining for canonical M1 and M2 markers on cardiac tissue to analyze the possibility of CA in regulating macrophage polarization. As shown in Fig. 2G, both M1 (CD86+) and M2 (CD206+) macrophages were increased in myocardium with MIRI compared to healthy myocardium (Fig. 2G), which were consistent with the results of flow cytometry. More importantly, CA reduced the M1 (CD86+)/M2 (CD206+) macrophage ratio (Fig. 2G). This implies that CA may participate in macrophage polarization regulation.

To further validate the effects of CA on pro-inflammatory cytokines and macrophage phenotype, we performed in vitro experiments. In agreement with the in vivo findings, CA suppressed IL-6 and TNF-α mRNA expression in RAW 264.7 macrophages (Fig. 2H). Because iNOS and Arg1 are key enzymes in inflammatory regulation—iNOS promoting pro-inflammatory responses and Arg1 supporting anti-inflammatory program [27]—we also assessed their expression. CA downregulated iNOS while upregulating Arg1 (Fig. 2H). Together, these data indicate that CA exerts anti-inflammatory effects during MIRI, which may be achieved by regulating the phenotypic transformation of macrophages.

### Caffeic acid suppressed inflammation-mediated cardiac fibroblast activation

**Fig. 3:**
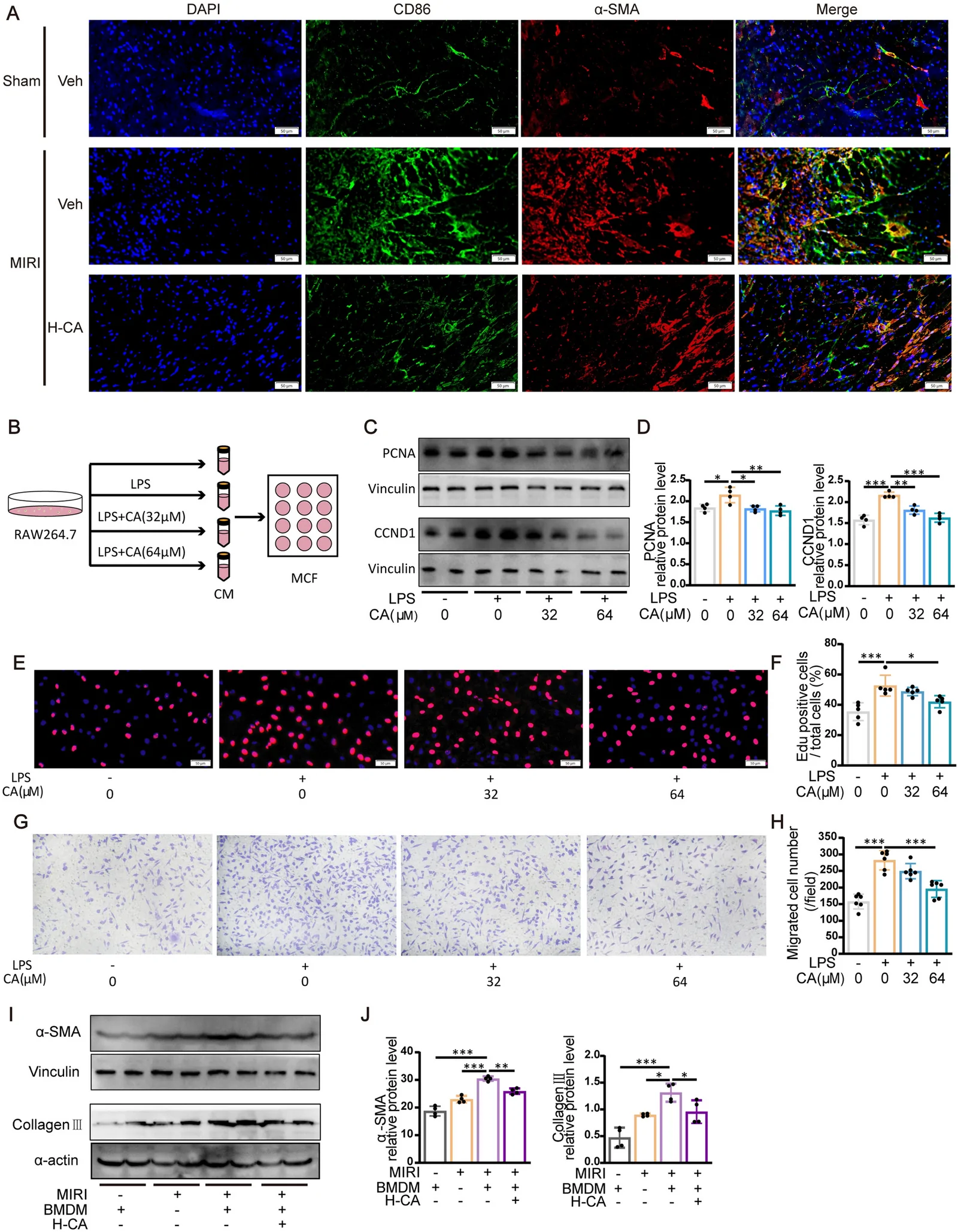
Caffeic acid mitigates inflammation-induced activation of myocardial fibroblasts. **A** Immunofluorescence staining for CD86 and α-SMA on mouse myocardial tissues. Green, CD86; red, α-SMA; blue, cell nuclei. Scale bar = 50 μm; n = 3 mice per group. **B** Schematic of the experimental procedure. The CM was collected from RAW 264.7 cells after culture under different conditions: Control, LPS, LPS+CA (32 μM), LPS+CA (64 μM). This CM was then applied to cardiac fibroblast cultures to assess its effects on proliferation and migration. **C**, **D** Representative images and quantification of western blot analysis to detect the protein levels of PCNA and CCND1 in MCFs. Vinculin served as a loading control. n = 4 independent biological replicates per group. **E** EdU assay for evaluating the proliferation rate of MCFs. Red, proliferating cells; blue, cell nuclei. Scale bar = 50 μm. **F** EdU-positive rate quantification. In randomly selected view fields, the number of EdU-positive cells (red) and the total number of cells (blue + red) were counted using ImageJ software. The ratio of EdU-positive cells to total cells was then calculated. n = 5 independent biological replicates per group. **G** Migratory activation of MCFs assessed using transwell migration assay. **H** Quantification of migrated cells was performed by counting the cell number in randomly selected fields using ImageJ software. n = 6 independent biological replicates per group. **I**, **J** Representative images and quantification of western blot analysis to detect the protein levels of α-SMA and collagen III in cardiac tissue. Vinculin and α-actin served as loading controls. n = 4 mice per group. *P < 0.05, **P < 0.01, ***P < 0.001. Statistical analyses were performed using one-way ANOVA followed by Tukey's post hoc test. Results are presented as mean ± SD

Macrophage–fibroblast crosstalk is a recognized driver of fibrogenesis, whereby inflammatory macrophage subsets promote fibroblast activation, myofibroblast differentiation, and TGF-β–dependent pro-fibrotic programs [28]. Initially, dual immunofluorescence staining for CD86 and α-SMA was conducted on murine myocardial sections to evaluate their spatial co-localization (Fig. 3A). Notably, both markers exhibited high expression in the infarct border zone of the MIRI+veh group, while CA treatment decreased their expression, confirming CA's intervention in inflammation–fibrosis crosstalk. To assess the contribution of macrophage-driven inflammatory cues to fibroblast activation and the effect of CA, we collected conditioned medium (CM) from RAW 264.7 cells treated with LPS with or without CA and examined fibroblast proliferative responses (Fig. 3B). We next evaluated the cell cycle–related proteins PCNA and CCND1 by western blot. LPS stimulation increased PCNA and CCND1 protein levels, whereas CA treatment attenuated these increases (Fig. 3C, D), consistent with the corresponding mRNA changes in PCNA and CCND1/2/3 (Fig. S1A). EdU staining further showed that LPS-containing CM increased the fraction of EdU-positive fibroblasts, and this effect was suppressed by CA co-treatment (Fig. 3E, F). To assess migration, transwell assays were performed to quantify fibroblast migratory responses to CM from LPS- or CA-treated macrophages: CM from LPS-stimulated macrophages promoted fibroblast migration, whereas CM from CA-treated macrophages abrogated this effect (Fig. 3G, H). Consistently, wound-healing assays showed accelerated wound closure at 12 h following exposure to CM from LPS-stimulated macrophages, while CM from CA-treated macrophages significantly inhibited wound closure (Fig. S1B–C). Collectively, these co-culture data indicate that CA suppresses inflammation-driven fibroblast proliferation and migration in vitro. To conclusively determine whether M1 macrophages aggravate myocardial fibrosis in MIRI mice, we conducted intramyocardial adoptive transfer of polarized BMDM and assessed the expression of fibrotic markers. The results showed that BMDM promoted the expression of α-SMA and collagen III in the setting of MIRI, whereas CA administration significantly inhibited this effect (Fig. 3I–J).

Nevertheless, it remains unclear whether CA exerts a direct inhibitory effect on fibroblasts. We performed an in vitro experiment in which cardiac fibroblasts were directly stimulated with TGF-β1 ± CA. CA did not significantly inhibit MAPKs/CREB activation (Fig. S1D–E) nor reduce α-SMA or collagen III levels (Fig. S1F–G) in fibroblasts. It follows that CA does not directly suppress fibroblast activation in the absence of inflammatory cells under these tested conditions.

### Caffeic acid alleviated macrophage-induced inflammation and myocardial fibrosis via MAPK pathway

**Fig. 4:**
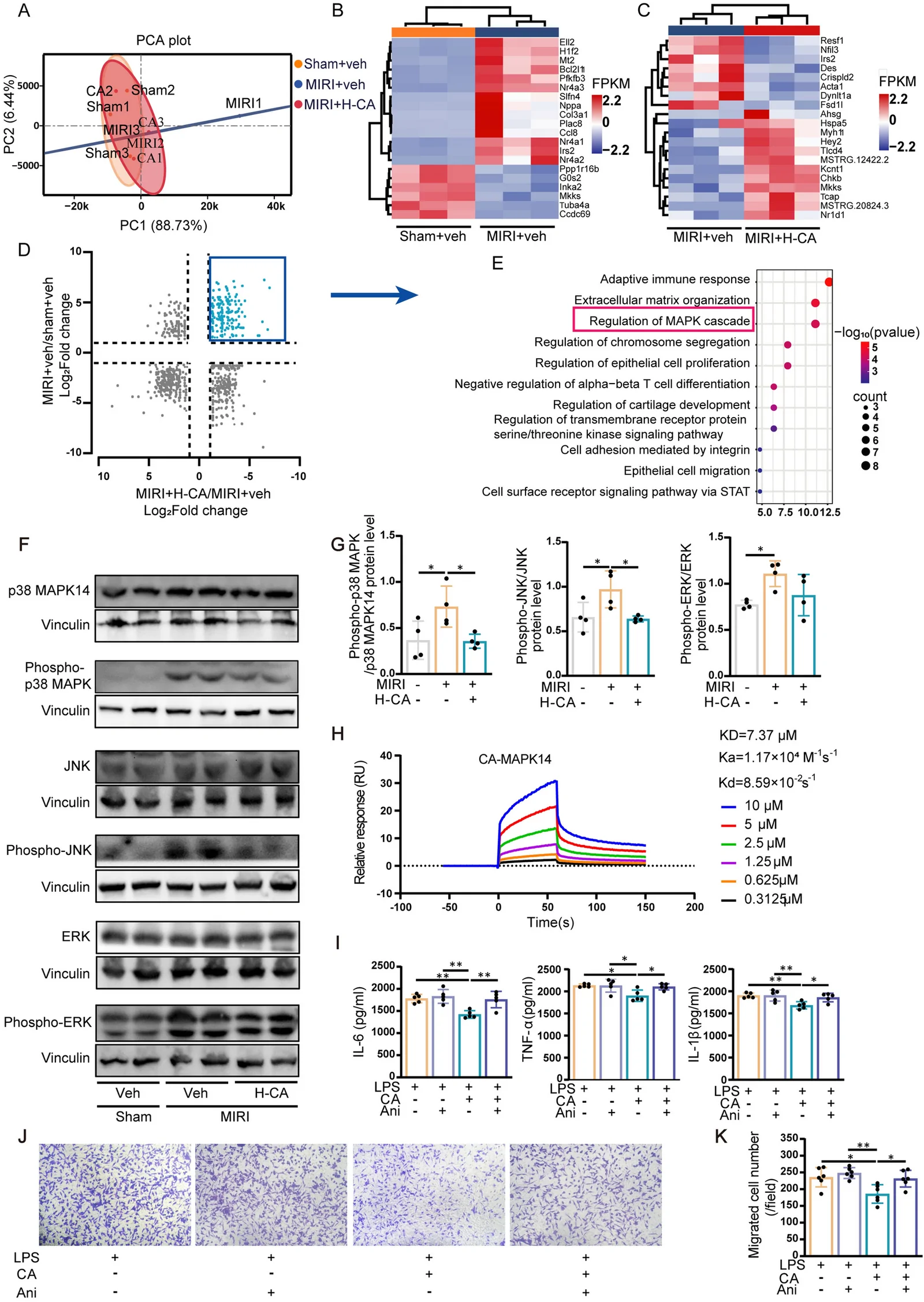
The effects of CA on macrophage-induced inflammation and myocardial fibrosis via MAPKs. **A** Principal component analysis (PCA) confirming similar response phenotypes between sham+veh and MIRI+CA group that was partially distinct from those in the MIRI+veh group. n = 3 mice per group. **B**, **C** Illustration of gene differential expression between MIRI+veh versus sham+veh groups and MIRI+H-CA versus MIRI+veh groups, respectively. n = 3 mice per group. **D** Scattered plot showing the differentially expressed genes that overlapped between MIRI+veh versus sham+veh groups and MIRI+H-CA versus MIRI+veh groups. n = 3 mice per group. **E** Bubble plots showing the signaling pathways of the differentially expressed genes (marked with blue in panel **D**) identified by KEGG. **F**, **G** Representative immunoblots of p38 MAPK14, phospho-p38 MAPK, JNK, phospho-JNK, ERK, and phospho-ERK in myocardial tissue of mice. Each protein level was assessed (Vinculin as loading control), and statistical comparisons were made for the phospho-p38 MAPK/p38 MAPK14, phospho-JNK/JNK and phospho-ERK/ERK ratios, respectively. n = 4 mice per group. **H** SPR analysis showing the binding affinity between CA and p38 MAPK14. The sensorgram was globally fitted to a 1:1 Langmuir binding model to determine the association rate constant (ka), dissociation rate constant (kd), and dissociation constant (KD). **I** The content of IL-6, TNF-α, and IL-1β in the cell supernatant of RAW 264.7 cells. n = 5 independent biological replicates per group. **J** Representative images of the transwell migration assay for MCFs. **K** Quantitative analysis of migrated cells per view field. n = 6 independent biological replicates per group. *P<0.05, **P<0.01. One-way ANOVA followed by Tukey's post hoc test was used in panel G, I and K. Data are presented as mean ± SD

To further elucidate the mechanisms underlying the therapeutic effects of CA, high-throughput RNA sequencing (RNA-seq) was performed for transcriptome profiling. Principal component analysis (PCA) revealed tight clustering of sham+veh and MIRI+CA samples, but the MIRI+veh group was heterogeneous: MIRI1 separated along PC1, whereas MIRI2 and MIRI3 lay close to the other groups (Fig. 4A). Considering the small sample size (n = 3 per group), the heterogeneity within the MIRI+veh group and the PCA separation were sensitive to a single sample. These data should therefore be interpreted as indicating a partial, rather than complete, transcriptomic shift after CA treatment. Differentially expressed genes (DEGs) were visualized in heat maps (Fig. 4B, C), showing strong within-group similarity across replicates. We next identified overlapping DEGs between the MIRI+CA versus MIRI+veh comparison and the MIRI+veh versus sham+veh comparison (Fig. 4D). We then focused on genes that were upregulated in MIRI+veh versus sham+veh but downregulated in MIRI+CA versus MIRI+veh (blue sector in Fig. 4D). KEGG enrichment analysis of this gene subset indicated that CA treatment downregulated pathways related to adaptive immune responses, ECM organization, MAPK cascade regulation, and so on (Fig. 4E). Given the small sample size and the heterogeneity within groups, the RNA-seq findings should be regarded as exploratory and require validation in a larger cohort.

We subsequently investigated the MAPK signaling cascade in greater detail. Previous studies have shown that p38 MAPK activation contributes to cardiovascular disease through multiple mechanisms, including enhancement of cardiac fibrosis via increased TNF-α and IL-6 release in cardiomyocytes, promotion of fibroblast differentiation, and stimulation of TGF-β production in cardiac fibroblasts [11, 16]. We therefore assessed whether CA interferes with MAPK signaling in our model. Western blot analysis revealed increased phosphorylation of three key MAPK components—p38 MAPK, JNK, and ERK—in cardiac tissue from MIRI mice, consistent with MAPK activation. CA treatment attenuated this phosphorylation, with particularly pronounced suppression of phospho-p38 MAPK and phospho-JNK (Fig. 4F, G).

We performed molecular docking to predict the potential for direct binding between CA and MAPK. CA binds to MAPK and engages in multiple hydrogen-bonding interactions with key residues, particularly SER-293 and SER-251, suggesting a stable binding mode that may competitively inhibit MAPK phosphorylation (Fig. S2A). Subsequently, to characterize the CA–p38 MAPK interaction, SPR analysis was performed. Global fitting of the sensorgrams to a 1:1 Langmuir binding model revealed dose-dependent binding of CA to p38 MAPK. The kinetic analysis showed a fast association and fast dissociation profile, characteristic of small-molecule–protein interactions, with a dissociation constant (KD) of 7.37 μM (Fig. 4H). Because ligand binding can stabilize specific conformational states of target proteins and thereby alter their biophysical properties, including thermal stability and proteolytic resistance [29], we next performed a cellular thermal shift assay (CETSA) followed by western blotting to confirm the target engagement of CA to p38 MAPK. In the DMSO control group, p38 MAPK14 degradation began at 45 °C, whereas degradation at the same temperature was markedly reduced in the CA-treated group (Fig. S2B–C).

LPS stimulation induces MAPK phosphorylation, activates downstream signaling, and promotes the production of pro-inflammatory mediators such as IL-6, TNF-α, and IL-1β [9], which was confirmed in our study (Fig. 4I). CA reduced LPS-induced secretion of IL-6, TNF-α, and IL-1β; however, this inhibitory effect was reversed by the MAPK agonist anisomycin (Ani) (Fig. 4I), indicating that CA may suppress inflammation by targeting MAPKs. Consistently, transwell migration assays showed that anisomycin reversed the CA-mediated inhibition of MCF migration when fibroblasts were exposed to the CM from LPS-treated macrophages (Fig. 4J, K). Collectively, these findings demonstrate that CA mitigates macrophage-driven inflammation and the resulting fibroblast migration by inhibiting MAPK signaling.

To investigate the participation of MAPK in the beneficial effects of CA on post-MIRI pathology, we performed in vivo validation using cardiomyocyte-specific MAPK14-knockout mice. The findings showed that CA conferred comparable protection against MIRI-induced cardiac dysfunction (Fig. S2D–E), myocardial fibrosis (Fig. S2F–G), and macrophage polarization (Fig. S2H) to that achieved by MAPK14 ablation, corroborating our hypothesis that CA exerts its anti-inflammatory and anti-fibrotic effects in MIRI, at least in part, via MAPK suppression.

### Caffeic acid suppressed inflammation and cardiac fibroblast activity via the MAPKs/CREB pathway in vitro

**Fig. 5:**
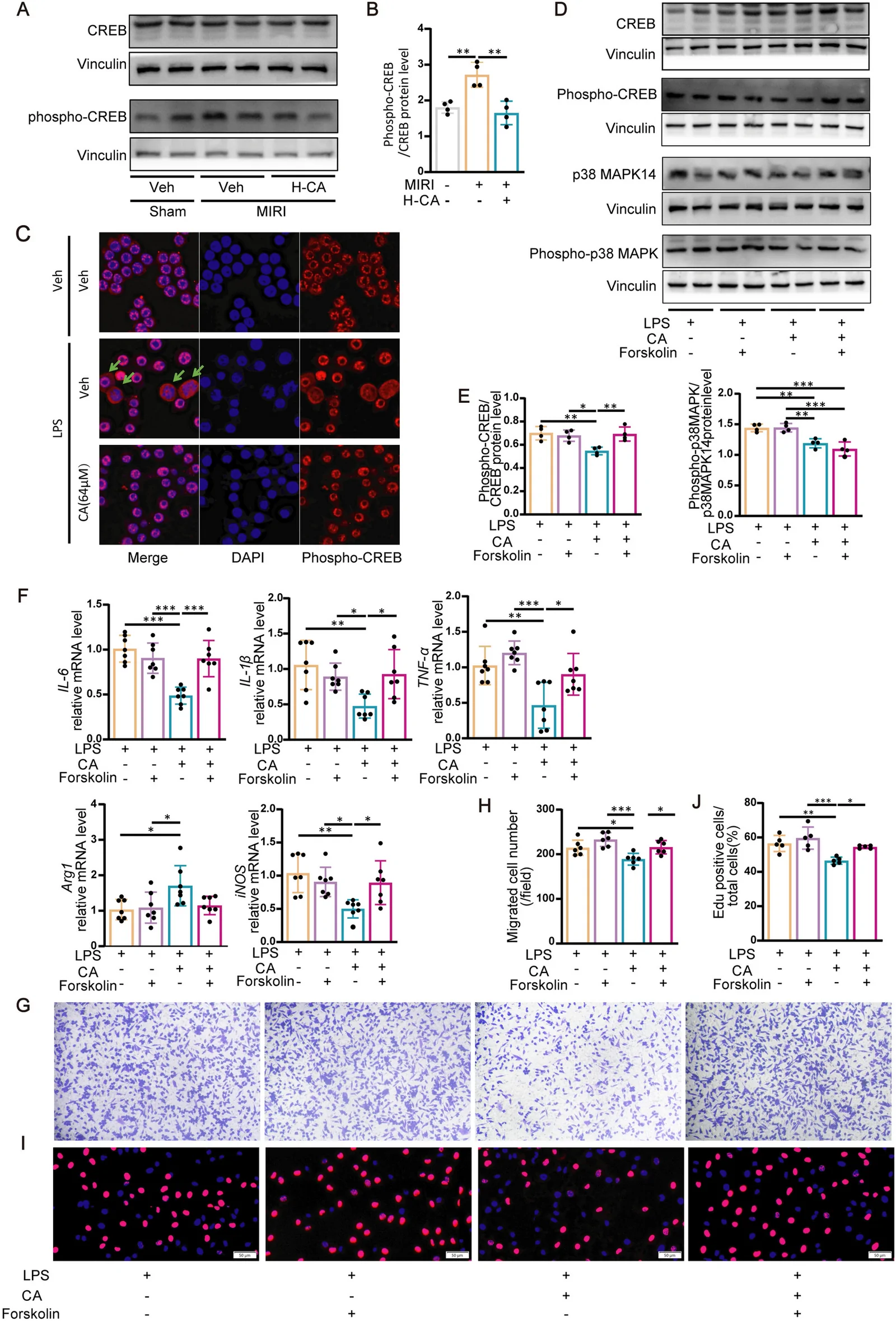
Effects of CA on inflammation and cardiac fibroblast activity via the MAPKs/CREB pathway. **A**, **B** The protein expression of CREB and phospho-CREB in cardiac tissues determined by western blot analysis. Protein levels of phospho-CREB and CREB were assessed (Vinculin as loading control), and statistical comparison was made for the phospho-CREB/CREB ratio. n = 4 mice per group. **C** Representative immunofluorescence images showing subcellular localization of phospho-CREB in RAW 264.7 cells treated with vehicle or CA (64 μM) in the presence of LPS or not. Red, phospho-CREB; blue, cell nuclei. n = 3 independent biological replicates per group. **D**, **E** Western blot analysis for detecting CREB, phospho-CREB, p38 MAPK14, and phospho-p38 MAPK in RAW 264.7 cells treated with forskolin or CA in the presence of LPS. Vinculin served as a loading control. n = 4 independent biological replicates per group. **F** Relative mRNA levels of IL-6, IL-1β, TNF-α, Arg1, and iNOS in RAW 264.7 following forskolin or CA (64 μM) treatment. n = 7 independent biological replicates per group. **G**, **H** Representative images of transwell migration assay and quantitative analysis of migrated cell numbers. n = 6 independent biological replicates per group. **I**, **J** EdU assay for determining the proliferation rate of MCFs. Red, proliferating cells; blue, cell nuclei. Scale bar=50 μm; n = 5 independent biological replicates per group. *P < 0.05, **P < 0.01, ***P < 0.001. Statistical analyses were performed using one-way ANOVA followed by Tukey's post hoc test. Results are presented as mean ± SD

Activation of p38 MAPK signaling enhances its downstream effector CREB, thereby promoting cardiac inflammation and remodeling [16]. We therefore examined whether CA exerts protective effects through CREB. Western blot analysis showed that phospho-CREB levels were significantly elevated in MIRI+veh mice compared with controls, whereas CA treatment reversed this increase (Fig. 5A, B). These findings were corroborated by immunofluorescence staining, which showed a strong phospho-CREB signal in the LPS+veh group that was markedly suppressed by CA treatment (Fig. 5C).

To further probe the role of CREB signaling, the CREB agonist forskolin was applied in vitro. Forskolin counteracted the inhibitory effect of CA on phospho-CREB expression but did not reverse CA-mediated suppression of phospho-p38 MAPK (Fig. 5D, E), supporting the involvement of the MAPKs/CREB signaling cascade.

We next assessed inflammatory mediator expression in RAW 264.7 cells. RT-qPCR showed that CA attenuated LPS-induced upregulation of IL-6, IL-1β, TNF-α, and iNOS and reversed the LPS-induced reduction of Arg1. Notably, the anti-inflammatory effect of CA was attenuated by forskolin (Fig. 5F). Furthermore, in the RAW 264.7 conditioned medium system, CA reduced the migratory and proliferative capacities of MCFs, whereas forskolin enhanced these activities, as shown by transwell migration assays (Fig. 5G, H) and EdU assays (Fig. 5I, J). Taken together, these results indicate that CA inhibits inflammation and attenuates the proliferative and migratory activities of MCFs through the MAPKs/CREB pathway in vitro.

### Caffeic acid ameliorated myocardial fibrosis via the MAPKs/CREB signaling pathway in MIRI mice

**Fig. 6:**
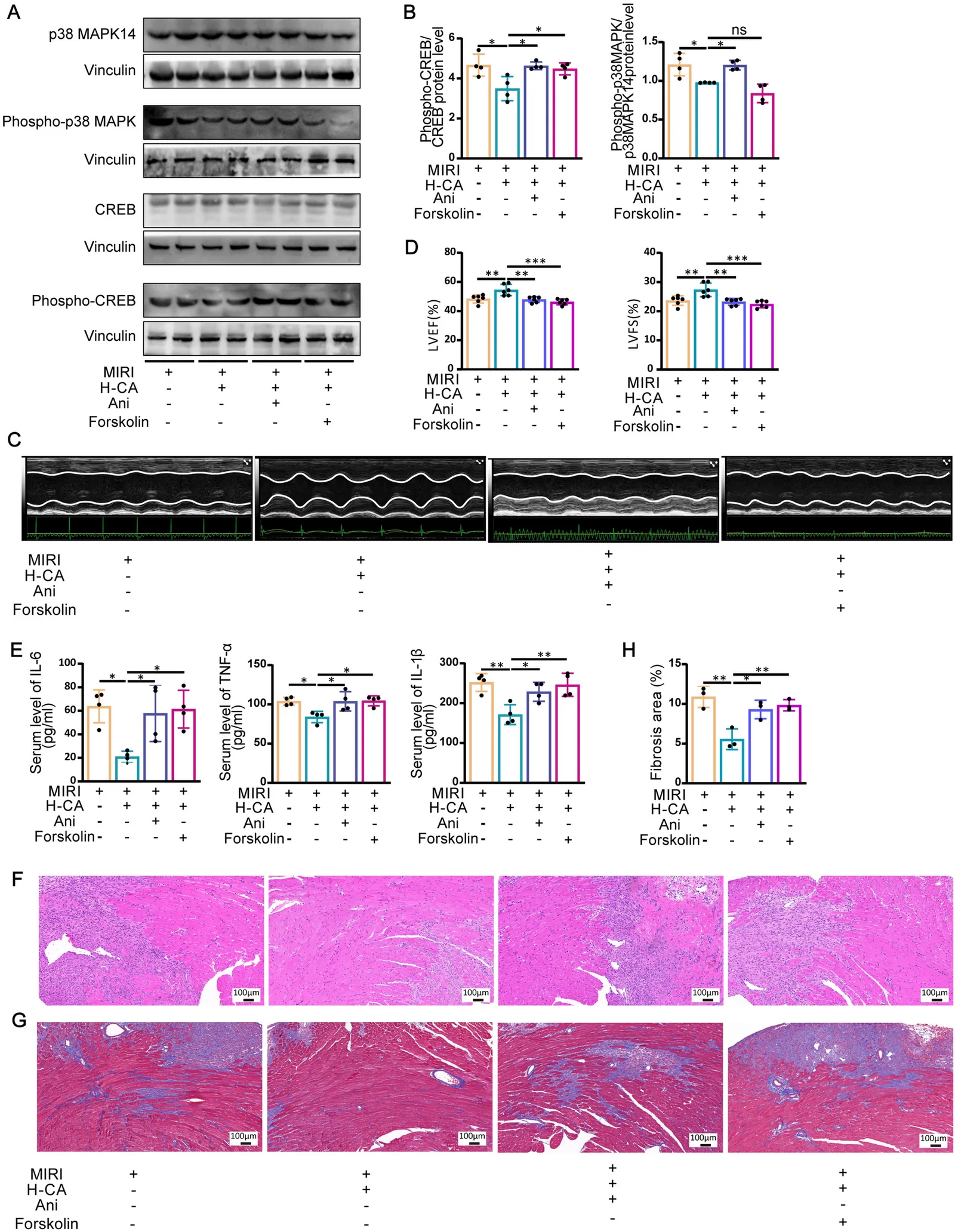
The roles of MAPKs and CREB in the amelioration of inflammation and myocardial fibrosis by CA. **A**, **B** Western blot analysis used to detect the protein expression of CREB, phospho-CREB, p38 MAPK14, and phospho-p38 MAPK in the myocardial tissues of mice. Vinculin served as a loading control. n = 4 mice per group. **C**, **D** Representative images and quantitative analysis of echocardiography to reflect the cardiac function of mice. n = 6 mice per group. **E** ELISA for evaluating the serum contents of IL-6, IL-1β, and TNF-α. n = 4 mice per group. **F** H&E staining of myocardial tissues from mice. View fields were randomly selected in the infarct border zone. Scale bar = 100 μm; n = 3 mice per group. **G**, **H** Representative images and quantitative analysis of Masson's trichrome staining in myocardial tissue of mice. Red, myofibers; blue, collagen. Scale bar = 100 μm; n = 3 mice per group. *P < 0.05, **P < 0.01, ***P < 0.001. Statistical analyses were performed using one-way ANOVA followed by Tukey’s post hoc test. Results are presented as mean ± SD

Finally, to test whether CA ameliorates inflammation-associated myocardial fibrosis in MIRI mice through the MAPKs/CREB axis, we performed in vivo pharmacological validation by combining CA treatment with pathway activators targeting MAPK or CREB signaling. First, western blot analysis showed that anisomycin (Ani), used here as a pharmacological activator of MAPK signaling, attenuated the inhibitory effects of CA on phospho-p38 MAPK and phospho-CREB levels (Fig. 6A, B). In contrast, the CREB activator forskolin reversed CA-mediated suppression of phospho-CREB but did not significantly reverse CA-mediated inhibition of phospho-p38 MAPK (Fig. 6A, B), consistent with the in vitro findings (Fig. 5D, E).

Second, both anisomycin and forskolin attenuated the protective effect of CA on cardiac function, as reflected by reduced LVEF and LVFS on echocardiography (Fig. 6C, D). To further evaluate whether CA suppresses inflammation via MAPK/CREB signaling in vivo, we measured serum inflammatory cytokines and assessed myocardial histopathology. Both activators reversed the inhibitory effects of CA on serum IL-6, IL-1β, and TNF-α levels (Fig. 6E) and diminished the CA-associated improvement in histopathological changes (Fig. 6F).

Finally, Masson’s trichrome staining was performed to determine whether the anti-fibrotic effect of CA is mediated through MAPKs/CREB signaling. Consistent with the above findings, CA alleviated myocardial fibrosis, whereas co-administration of anisomycin or forskolin exacerbated fibrotic remodeling compared with CA treatment alone (Fig. 6G, H). Collectively, these in vivo data support that CA mitigates inflammation and myocardial fibrosis in MIRI mice, with effects that are sensitive to pharmacological activation of MAPK and CREB signaling.

## Discussion

Caffeic acid (CA) is a natural polyphenol widely present in the human diet (e.g., coffee, tea, red wine, fruits, and herbs) and exerts anti-inflammatory, antioxidant, and antibacterial activities [30]. Consistent with these activities, protective effects of CA and its analogs have been reported in cardiovascular contexts, including hyperglycemia-induced endothelial dysfunction [31], hypertension [32, 33], myocardial infarction [34], and MIRI [30]. Moreover, extensive in vivo and in vitro studies indicate that CA can attenuate fibrotic progression across multiple tissue and organ models by modulating key signaling pathways, suppressing pro-fibrotic mediators, and alleviating inflammation and oxidative stress [34–36], supporting its therapeutic promise against myocardial fibrosis. In the present study, we validated the efficacy of CA in a murine MIRI model and observed comprehensive cardioprotective effects, including improved cardiac function, attenuated hypertrophy, and reduced myocardial fibrosis. Building on these findings, we further investigated the pharmacological mechanisms underlying CA's actions using both in vivo and in vitro approaches. It is noteworthy that while CA derivatives generally exhibit improved pharmacokinetic profiles, the intrinsic activity of the parent compound remains a critical benchmark. Therefore, we first investigated unmodified CA in the context of inflammation-induced cardiac fibroblast activation, as its natural form is more directly relevant to dietary exposure and early-stage intervention. Demonstration of its efficacy in this setting further strengthens the rationale for future development of chemically optimized derivatives. Importantly, fibroblast activation represents an early cellular response that can be modeled in vitro within hours to days, whereas myocardial fibrosis reflects the cumulative tissue-level ECM deposition that requires a longer in vivo time course to become fully manifest. Our experimental design captures both levels, with in vitro assays addressing the activation phenotype and in vivo histology assessing established fibrosis.

In the dysregulated fibrotic process induced by MIRI, immune-cell infiltration and inflammatory cytokines exert pivotal effects [2, 37, 38]. Pro-inflammatory mediators promote M1 macrophage polarization, establishing a positive feedback loop that amplifies inflammation and myocardial injury, whereas anti-inflammatory cues support M2 polarization and protective feedback programs [39]. M1-driven inflammation and fibrosis have been implicated as major contributors of left ventricular dilation and subsequent heart failure in cardiovascular disease [2]. In our study, CA reduced inflammatory infiltration in the myocardium of MIRI mice, decreased inflammatory cytokines (IL-1β and TNF‑α), and lowered macrophage abundance, with a concomitant reduction in M1 polarization. Consistently, in RAW 264.7 macrophages, CA suppressed IL-6, TNF-α, and iNOS mRNA while promoting Arg1 expression, supporting a macrophage-mediated anti-inflammatory effect of CA. For in vivo validation, co-immunostaining for the inflammatory marker CD86 and the fibrotic marker α-SMA in myocardial tissues, along with intramyocardial adoptive transfer of macrophages, confirmed the link between M1 macrophages and subsequent myocardial fibrosis, and further demonstrated that CA suppresses this pathological process.

Within the MIRI microenvironment, immune-cell infiltration can trigger excessive fibroblast activation and proliferation via pro-fibrotic cytokines, driving ECM deposition and collagen secretion, ultimately contributing to adverse remodeling and contractile dysfunction [2, 37, 38]. Fibroblasts and macrophages engage in dynamic bidirectional crosstalk that shapes the local tissue microenvironment [40], and inflammatory macrophage subsets can drive fibroblast activation, myofibroblast differentiation, and TGF-β–dependent pro-fibrotic pathways [28]. Here, we applied a co-culture system using activated RAW 264.7 macrophages and mouse cardiac fibroblasts (MCFs) to evaluate whether CA mitigates macrophage-driven fibroblast activity. Consistent with prior concepts, our data provide evidence that inflammatory macrophage-derived cues promote fibroblast proliferation and migration. Importantly, CA inhibited this pathogenic interaction, indicating therapeutic potential for limiting myocardial fibrosis following MIRI.

To further define the cardioprotective mechanisms of CA, we performed RNA sequencing and found that genes upregulated in MIRI versus control and downregulated after CA treatment were enriched in the MAPK signaling cascade, providing a mechanistic direction for subsequent analyses. MAPKs, including ERK, JNK, and p38 MAPK, are central regulators of inflammatory signaling [9, 10], and their activation contributes to inflammatory gene expression as well as cardiac fibrosis and hypertrophy [41]. JNK and p38 MAPK, in particular, regulate inflammation, apoptosis, and fibrosis and promote expression of inflammatory mediators such as TNF-α, IL-1β, and IL-6, thereby adversely affecting cardiac function [42, 43]. MAPK-dependent macrophage polarization can also exacerbate cardiac injury through intercellular crosstalk [44]. In our study, CA reduced phosphorylation within the MAPK cascade, with prominent suppression of p38 MAPK and JNK. Integrating molecular docking predictions, SPR biophysical analysis, and CETSA target engagement validation, we obtained evidence supporting direct binding of CA to p38 MAPK. Furthermore, anisomycin, used here as a pharmacological activator of MAPK signaling, attenuated the inhibitory effects of CA on inflammation and cardiac fibroblast migration, supporting the involvement of MAPKs in this process. In the MIRI model, CA conferred cardioprotective effects comparable to those achieved by cardiomyocyte‑specific MAPK14 ablation, corroborating that MAPK suppression likely represents a major mechanism by which CA ameliorates MIRI‑related pathology.

Activation of p38 MAPK can further enhance downstream CREB activity, thereby promoting cardiac inflammation and remodeling [16]. CREB is a nuclear transcription factor that regulates genes involved in proliferation, differentiation, survival, and adaptive responses [45, 46]. Phosphorylated CREB is widely recognized as the active form of this transcription factor [46], and its phosphorylation at Ser133 is controlled by diverse kinases in a context-dependent manner [45]. CREB activation is linked to inflammatory responses [46, 47], including via MAPKs/CREB signaling [48, 49]. In our study, phosphorylated CREB was elevated after MIRI and was reduced by CA treatment, indicating CREB activation in the MIRI model and effective suppression by CA. Moreover, pharmacological activation of CREB attenuated the regulatory effects of CA on inflammatory mediators, fibroblast activity, cardiac function, and pathological tissue changes, supporting CREB involvement in the therapeutic mechanism of CA. Notably, CREB activation did not reverse CA-mediated inhibition of MAPK phosphorylation, whereas MAPK activation counteracted the suppression of CREB, consistent with a MAPKs/CREB signaling cascade.

## Conclusion

In this study, administration of the natural polyphenol caffeic acid (CA) in a murine MIRI model alleviated MIRI-induced cardiac dysfunction, pathological hypertrophy, and myocardial fibrosis. Modulation of the inflammatory microenvironment contributed to these cardioprotective effects, as evidenced by reduced macrophage infiltration and suppression of pro-inflammatory mediators. Mechanistically, the anti-inflammatory and anti-fibrotic actions of CA were mediated, at least in part, through inhibition of MAPKs/CREB signaling. Collectively, these findings support CA as a potential therapeutic candidate for limiting adverse fibrotic remodeling after MIRI.

This study has several limitations. First, additional mechanisms may contribute to CA-mediated regulation of fibrotic remodeling after MIRI and require further investigation. Second, the use of cell lines rather than primary cells may not fully recapitulate the in vivo pathological microenvironment. Third, although in vitro experiments confirmed that CA exerts no significant direct inhibitory effect on fibroblasts, the in vivo validation of the MAPKs/CREB pathway relied on pharmacological modulation without tissue- or cell-type–specific targeting; therefore, the relative contributions of macrophages and fibroblasts to the observed in vivo effects remain to be determined. Future studies using cell-type–specific approaches will be required to establish the causal roles of these populations in vivo.

## Supplementary Information

Supplementary material 1

## Data Availability

All data generated or analyzed during this study are included in this manuscript (and its Supplementary Information files).

## Abbreviations

BMDM: Bone marrow-derived macrophage
CA: Caffeic acid
CCND1/2/3: Cyclin D1/2/3
CM: Conditioned medium
CREB: CAMP-response element binding protein
DAMP: Damage-associated molecular pattern
ECM: Extracellular matrix
ERK: Extracellular signal-regulated kinase
GO: Gene ontology
JNK: C-Jun N-terminal kinase
KEGG: Kyoto Encyclopedia of Genes and Genomes
LAD: Left anterior descending coronary artery
LPS: Lipopolysaccharide
LVEF: Left ventricular ejection fraction
LVFS: Left ventricular fractional shortening
MAPK: Mitogen-activated protein kinase
MCF: Mouse cardiac fibroblast
M-CSF: Macrophage-colony-stimulating factor
MIRI: Myocardial ischemia reperfusion injury
NF-κB: Nuclear factor kappa-B
PCNA: Proliferating cell nuclear antigen
SPR: Surface plasmon resonance
WGA: Wheat germ agglutinin
