# Supplementary material for: Caffeic acid mitigates myocardial fibrosis via suppressing MAPKs/CREB-mediated inflammation in myocardial ischemia–reperfusion injury mice
Source: Chin Med. 2026 Oct 29;21:233. doi: 10.1186/s13020-026-01562-2 (PMC13721419; doi:10.1186/s13020-026-01562-2)
Supplement: Supplementary file 1 [file 13020_2026_1562_MOESM1_ESM.docx]

**Fig. S1 The effect of CA on inflammation-induced MCF activation and the direct inhibitory effect of CA on MCFs.** (A) RT-qPCR performed to detect the levels of cell cycle-related genes in MCFs. n=6 independent biological replicates per group. (B) Wound healing assay used to assess the migration ability of MCFs; wound closure was captured at 0 and 12 h post-scratch. (C) Quantification analysis of wound closure rate. The wound closure area was determined by subtracting the 12h wound area from the initial (0h) area. The closure rate was derived by dividing this closure area by the initial wound area. n=4 independent biological replicates per group. (D-E) Western blot analysis used to detect the protein expression of CREB, phospho-CREB, p38 MAPK14, and phospho-p38 MAPK in MCFs. Vinculin served as a loading control. n=4 independent biological replicates per group. (F-G) Western blot analysis for detecting the protein expression of α-SMA and Collagen Ⅲ in MCFs. Vinculin and α-actin served as a loading control. n=4 independent biological replicates per group. *P<0.05, **P<0.01, ***P<0.001. Statistical analyses were performed using one-way ANOVA followed by Tukey's post hoc test. Results are presented as mean ± SD.

**Fig. S2. In silico and biophysical validation of CA–MAPK interaction and functional comparison of CA treatment versus MAPK14 ablation in MIRI mice.** (A) Molecular docking of MAPK and CA. Intermolecular interactions are indicated by dashed lines, with the corresponding interaction distances explicitly labeled for the critical residues. (B) CETSA for detecting the thermal stabilization of p38 MAPK14 protein in RAW 264.7 cells treated with DMSO or CA (64 μM) across a temperature gradient (40-70℃). Vinculin served as a loading control. (C) Quantification of the p38 MAPK14 protein thermal stability from (B). Band intensities were quantified using ImageJ software, and the relative protein residual at each temperature point was calculated. n=3 independent biological replicates per group. (D-E) Representative short-axis M-mode echocardiographic images and corresponding quantitative measurements of LVEF and LVFS. n=6 mice per group). (F-G) Representative images of Masson's trichrome staining of myocardial sections and quantitative analysis of fibrosis. Myofibers and collagen are shown in red and blue, respectively. Scale bar=100 μm. n=3 mice per group. (H) Representative immunofluorescence images of CD86 (green) and CD206 (red) expression in mouse cardiac tissue. Nuclei were counterstained with DAPI (blue). Scale bar=50 μm; n=3 mice per group. *P<0.05. Statistical analyses were performed using one-way ANOVA followed by Tukey's post hoc test. Results are presented as mean ± SD.
